# Early Detection of Pancreatic Intraepithelial Neoplasias (PanINs) in Transgenic Mouse Model by Hyperpolarized ^13^C Metabolic Magnetic Resonance Spectroscopy

**DOI:** 10.3390/ijms21103722

**Published:** 2020-05-25

**Authors:** Prasanta Dutta, Susana Castro Pando, Marilina Mascaro, Erick Riquelme, Michelle Zoltan, Niki M. Zacharias, Seth T. Gammon, David Piwnica-Worms, Mark D. Pagel, Subrata Sen, Anirban Maitra, Shayan Shams, Florencia McAllister, Pratip K. Bhattacharya

**Affiliations:** 1 Department of Cancer Systems Imaging, The University of Texas MD Anderson Cancer Center, Houston, TX 77054, USA; pdutta@mdanderson.org (P.D.); NMZacharias@mdanderson.org (N.M.Z.); stgammon@mdanderson.org (S.T.G.); dpiwnica-worms@mdanderson.org (D.P.-W.); mdpagel@mdanderson.org (M.D.P.); 2 Department of Clinical Cancer Prevention, The University of Texas MD Anderson Cancer Center, Houston, TX 77030, USA; LSCastro@mdanderson.org (S.C.P.); marilina.m25@gmail.com (M.M.); EMRiquelme@mdanderson.org (E.R.); MAZoltan@mdanderson.org (M.Z.); FMcAllister@mdanderson.org (F.M.); 3 Department of Urology, The University of Texas MD Anderson Cancer Center, Houston, TX 77030, USA; 4 Department of Translational Molecular Pathology, The University of Texas MD Anderson Cancer Center, Houston, TX 77030, USA; ssen@mdanderson.org; 5 Department of Pathology, The University of Texas MD Anderson Cancer Center, Houston, TX 77030, USA; AMaitra@mdanderson.org; 6 School of Biomedical Informatics, The University of Texas Health Science Center, Houston, TX 77030, USA; Shayan.Shams@uth.tmc.edu

**Keywords:** metabolic plasticity and PanINs progression, metabolic imaging, metabolic rewiring, kinetic rate constant and modeling, early detection, pancreatic cancer, hyperpolarization, MRS

## Abstract

While pancreatic cancer (PC) survival rates have recently shown modest improvement, the disease remains largely incurable. Early detection of pancreatic cancer may result in improved outcomes and therefore, methods for early detection of cancer, even premalignant lesions, may provide more favorable outcomes. Pancreatic intraepithelial neoplasias (PanINs) have been identified as premalignant precursor lesions to pancreatic cancer. However, conventional imaging methods used for screening high-risk populations do not have the sensitivity to detect PanINs. Here, we have employed hyperpolarized metabolic imaging in vivo and nuclear magnetic resonance (^1^H-NMR) metabolomics ex vivo to identify and understand metabolic changes, towards enabling detection of early PanINs and progression to advanced PanINs lesions that precede pancreatic cancer formation. Progression of disease from tissue containing predominantly low-grade PanINs to tissue with high-grade PanINs showed a decreasing alanine/lactate ratio from high-resolution NMR metabolomics ex vivo. Hyperpolarized magnetic resonance spectroscopy (HP-MRS) allows over 10,000-fold sensitivity enhancement relative to conventional magnetic resonance. Real-time HP-MRS was employed to measure non-invasively changes of alanine and lactate metabolites with disease progression and in control mice in vivo, following injection of hyperpolarized [1-^13^C] pyruvate. The alanine-to-lactate signal intensity ratio was found to decrease as the disease progressed from low-grade PanINs to high-grade PanINs. The biochemical changes of alanine transaminase (ALT) and lactate dehydrogenase (LDH) enzyme activity were assessed. These results demonstrate that there are significant alterations of ALT and LDH activities during the transformation from early to advanced PanINs lesions. Furthermore, we demonstrate that real-time conversion kinetic rate constants (k_PA_ and k_PL_) can be used as metabolic imaging biomarkers of pancreatic premalignant lesions. Findings from this emerging HP-MRS technique can be translated to the clinic for detection of pancreatic premalignant lesion in high-risk populations.

## 1. Introduction

Detection of pancreatic cancer (PC) at early stages remains a great challenge in clinical oncology. In contrast to the declines in cancer-related deaths from other malignancies, progress in the management of pancreatic ductal adenocarcinoma (PDAC) has been slow, and the incidence of cancer-related deaths due to PDAC continues to rise [1]. PDAC develops relatively symptom-free and is one of the leading causes of cancer-related deaths in the United States [2,3,4,5]. In 2020 alone, it is estimated that about 57,600 people (30,400 men and 27,200 women) will be diagnosed with pancreatic cancer and about 47,050 people (24,640 men and 22,410 women) will die of the disease [6]. Early detection of PDAC is unusual and typically incidental, with the majority (~85%) presenting with locally advanced or metastatic disease, when surgery, the only curative modality, is not an option. Overall, PDAC is associated with a dire prognosis and a 5-year survival rate of only 8% [7]. Despite these grim numbers, there is unequivocal evidence that diagnosis of PDAC at earlier, resectable stages has a profoundly favorable impact on prognosis. The 5-year survival of resected PDAC is as high as ~25%–30% in major treatment centers, increasing to 30%–60% for tumors < 2cm, and as high as 75% for “minute” lesions under 10 mm in size [8,9]. Thus, early detection of PDAC is an area of highest priority. The absence of early symptoms and lack of a reliable screening test have created a critical need for identifying and developing new non-invasive biomarkers for pancreatic cancer early detection [10]. Therefore, there is an urgency to develop novel methods for the detection of pancreatic cancer preneoplastic lesions.

Pre-invasive pancreatic intraepithelial neoplasia (PanINs) have been identified as precursor lesions of PC [11,12,13,14]. No current methods of screening in clinic can detect PanINs. While KRAS is the most common mutated gene (over 70%) in PDAC, mutations have also been detected in several other genes, such as TP53, SMAD4, CDKN2A, and GNAS [15,16]. It has been reported that genetic mutations, along with the unique tumor microenvironment in PDAC, are susceptible to metabolic plasticity [17]. There is an unmet need to develop capabilities to non-invasively detect PanINs prior to invasive stages of the disease where the prevention and treatment will be most effective.

Real-time hyperpolarized magnetic resonance spectroscopy (HP-MRS) has become an emerging imaging modality by providing valuable information on previously inaccessible aspects of biological processes by detecting endogenous, non-radioactive ^13^C-labeled molecules that can monitor enzymatic conversions in vivo through key biochemical pathways [18]. It allows over 10,000-fold sensitivity enhancement relative to conventional magnetic resonance and is a non-toxic method for assessing tissue metabolism and other physiologic properties [19]. To date, the most studied HP ^13^C compound is pyruvate, as it plays a central role in many biochemical and metabolic pathways [20] Therefore, pyruvate metabolism-based HP-MRS represents a potential methodology to identify and understand early metabolic changes, to enable detection of early-stage and advanced PanINs, as well as early PDAC, for which no methods of detection currently exist. A Phase I clinical trial employing HP [1-^13^C] pyruvic acid for the diagnosis of prostate cancer was concluded at the University of California, San Francisco; it demonstrated safety and feasibility in the clinic [21]. Recently, a pilot study reported the feasibility of HP [1-^13^C] pyruvate imaging in pancreatic cancer patients, and no adverse effect was observed after bolus injection of pyruvate [22].

The Warburg effect is a metabolic signature of many solid tumors that causes lactate production from pyruvate even in the presence of oxygen [23]. The rate of lactate production by upregulation of lactate dehydrogenase (LDH) has been revealed as a metabolic biomarker for cancer initiation and progression in multiple animal models [24,25,26]. Another metabolic pathway of interest from pyruvate to alanine via the enzyme alanine transaminase (ALT) can be interrogated after injecting hyperpolarized [1-^13^C] pyruvate. It was recently reported that the alanine/lactate concentration ratio, measured in PanINs and PDAC lesions, showed a significant decrease with disease progression, primarily due to an increase in lactate concentration, which was consistent with the increased fluorodeoxyglucose uptake [27]. Our group has recently reported that hyperpolarized [1-^13^C] pyruvate metabolic imaging is feasible in assessing aggressiveness in pancreatic cancer patient-derived xenograft (PDX) models [28].

In this current study, we performed a comprehensive metabolic imaging study applying hyperpolarized pyruvate imaging in genetically engineered mouse (GEM) models with progression of PanIN lesions. The study was designed to examine metabolic changes in the relevant GEM models, as the PanINs evolve with mouse age. This experimental methodology and consequently metabolic conversion kinetic parameters have been adopted to identify non-invasive surrogate imaging biomarkers. This approach may aid detection of premalignant pancreatic lesions or pancreatic cancer at the earliest resectable stages.

## 2. Results

### 2.1. Histology

**Figure 1:**
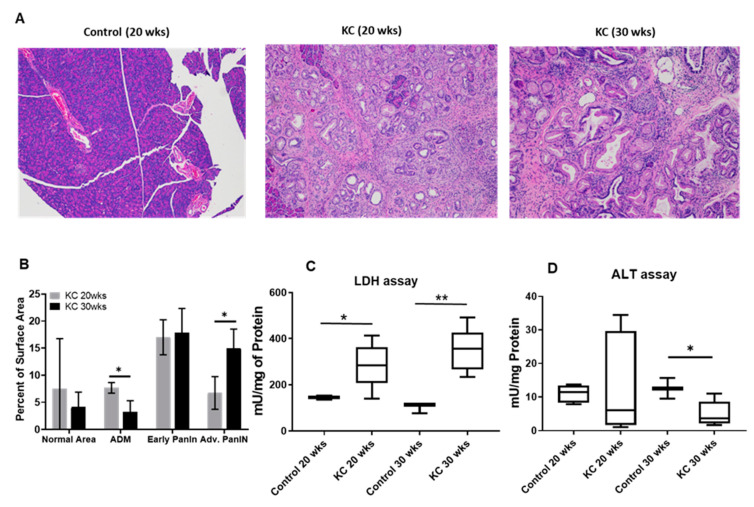
(**A**) Representative Hematoxylin and Eosin (H&E) micrographs for different mice groups. Histological staining demonstrates the differences in morphology and cellularity in normal pancreas (control 20 wks) and early- (P48:Cre; LSL-KRAS^G12D^—KC; 20 wks) and advanced-stage (KC 30 wks) pancreatic intraepithelial neoplasias (PanINs). (**B**) Lesion quantification as presented by the percent of surface area varied with PanINs progression from early to advanced stage. Normalized values of active alanine transaminase (ALT) and lactate dehydrogenase (LDH) enzyme concentrations for different mice groups (*n* =8) are shown in (**C**) and (**D**) respectively. Normal pancreas and early-stage PanINs demonstrate high concentration of ALT and low levels of LDH, whereas the advanced-stage PanINs show low concentration of ALT and high concentration of LDH measured in mU/mg unit. * means *p* < 0.05; ** means *p* < 0.001.

Genetically engineered mouse (GEM) models (P48:Cre; LSL-KRAS^G12D^ (KC)) with progression of PanIN lesions and control animals (P48:Cre or WT C57BL/6) without pancreatic lesions were employed in our study. We collected tissue samples from the different age groups of mice and investigated the evolution of disease with time by histology. Figure 1A displays Hematoxylin and Eosin (H&E) micrographs of control mice (WT 20 weeks), KC mice (20 weeks), and KC mice (30 weeks of age). The surface area of the lesions was quantified and is shown as mean % total area (+/− SEM; Figure 1B).

### 2.2. Active LDH and ALT Enzymes Assay

We performed an active LDH and ALT enzymes assay on the different PanINs tissues. We observed the active concentration of LDH and ALT enzymes remained the same for control mice at 20 and 30 weeks of age, whereas as in KC mice, the active LDH concentration significantly increased at 30 weeks compared to 20 weeks of age (*p* = 0.013). In contrast, the active ALT concentration decreased at 30 weeks for KC mice relative to 20 weeks (Figure 1C,D).

### 2.3. Ex Vivo ^1^H NMR Metabolomics Study

**Figure 2:**
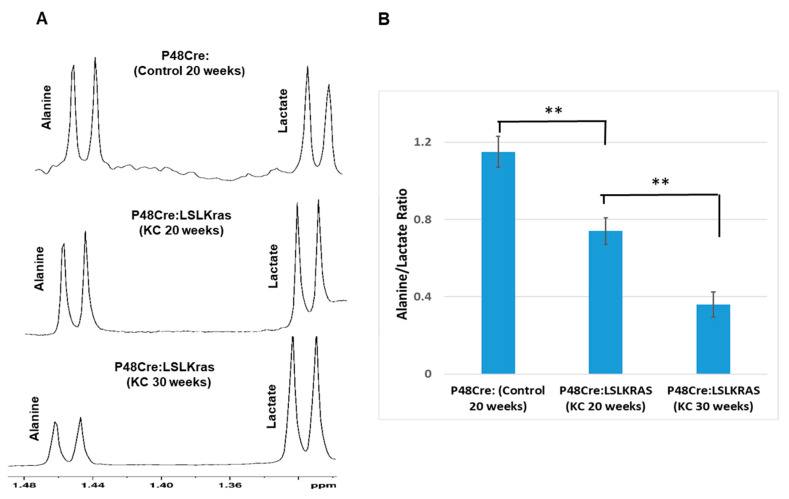
(**A**) Steady-state high-resolution nuclear magnetic resonance (^1^H- NMR) spectra of ex vivo tissue samples for different mouse groups (*n* = 10). The alanine and lactate peak intensity alters inversely with PanINs progression. For alanine, it decreases, but lactate peak intensity increases as the PanINs progress from early to advanced stage. (**B**) The net ratio of alanine to lactate (Alanine/Lactate) gradually reduces with PanINs progression to advanced stages. ** means *p* < 0.001.

We investigated the concentration changes of alanine and lactate as the PanINs progressed in KC mice and compared with pancreas of control mice using standard ex vivo NMR metabolomics analysis. Figure 2A depicts that highest alanine peak intensity is found in control mice which gradually decreased in KC mice as the PanINs progressed with age 20 and 30 weeks. In contrast, the lactate peak intensity increased in KC mice compared to control mice. The alanine/lactate ratio was plotted for different mouse groups and is presented in Figure 2B. The data revealed that the ratio was significantly decreased in advanced PanINs.

### 2.4. In Vivo ^13^C MRS of Pyruvate

**Figure 3:**
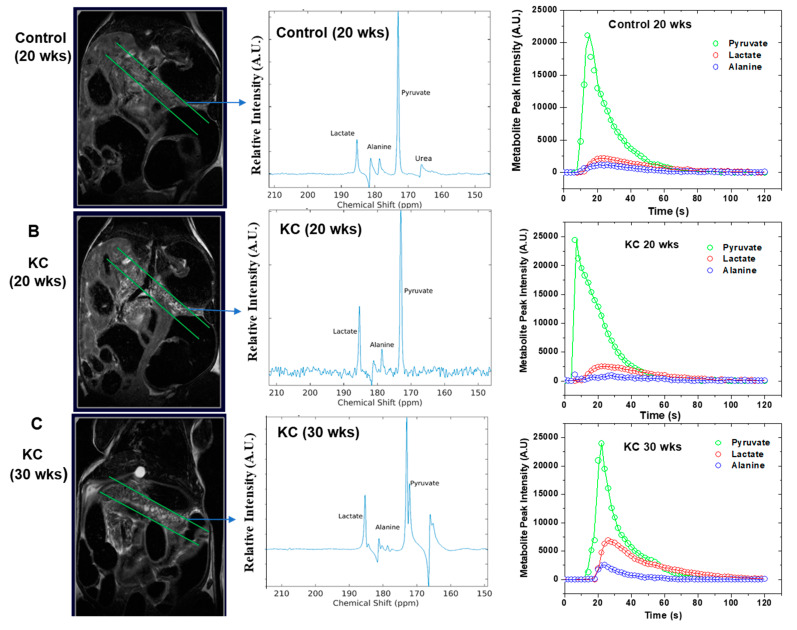
T_2_-weighted coronal MR image, in vivo ^13^C MR spectra (acquired from selected slabs) and normalized signal intensity of different metabolites presented for (**A**) control mice with 20 weeks of age, (**B**) KC mice with an age of 20 weeks, and (**C**) KC mice with an age of 30 weeks. MRI images depict the smaller number of nodules on early stage PanINs (**B**) and the number and the size of the nodules increase as the PanINs progress to advanced stages (**C**). The real-time ^13^C MR spectra were acquired in vivo following injection of hyperpolarized pyruvate. The lactate production is significantly higher in advanced PanINs. The time evolution of all three metabolites was captured up to two minutes, and the experimental data were fitted to unidirectional kinetic modelling (**C**) for apparent rate constants k_PA_ and k_PL_ determination. The fitted parameters are summarized in Table 1.

We employed high-resolution T_2-_weighted proton (^1^H) MRI to examine the normal pancreas tissue in control mice and the evolution of PanINs over time in KC mice. We saw normal pancreas morphology (Figure 3A) on MRI scans in control mice independent of age (10, 20, or 30 weeks). However, we observed prominent PanIN nodules by MRI from 20 weeks onwards (Figure 3B) for KC mice. We saw an increase in the number and size of PanIN nodules in all KC mice at 30 weeks, which we labelled as aggressive PanIN (Figure 3C).

We selected a slab with 4–5 mm thickness to acquire real-time ^13^C MRS in vivo to measure changes of alanine and lactate metabolites with disease progression compared to control mice, following injection of hyperpolarized [1-^13^C] pyruvate. There is always a possibility of admixing of some HP-MRS signal originating from normal tissue. Chances of such signal contamination is reduced with the progression of PanIN as PanIN nodules increase in size with advanced precancerous lesions. The alanine-to-lactate (Ala/Lac) signal intensity ratio was found decreased as the disease progressed from normal pancreas to low-grade PanINs and high-grade PanINs (Figure 3A–C). These results demonstrated that there were significant alterations of alanine and lactate production from injected hyperpolarized [1-^13^C] that favored the transformation of aggressive PanINs lesions.

### 2.5. Kinetic Modeling

We fitted the real-time metabolic conversion data to extract the kinetic rate constants, k_PA_ (pyruvate-to-alanine) and k_PL_ (pyruvate-to-lactate), applying the unidirectional model as shown in Figure 3C. The RStudio software (RStudio Inc., Boston, MA, USA) was used for kinetic modeling and fitting the experimental data. We briefly described our approach for fitting the experimental data in the Supplementary Materials section. The values of real-time conversion kinetic rate constants (k_PA_ and k_PL_) varied with PanINs progression (Table 1). Our results suggested that real-time conversion kinetic rate constants (k_PA_ and k_PL_) can be used as metabolic imaging biomarkers for assessing the early stage of pancreatic diseases.

### 2.6. Immunohistochemistry

**Figure 4:**
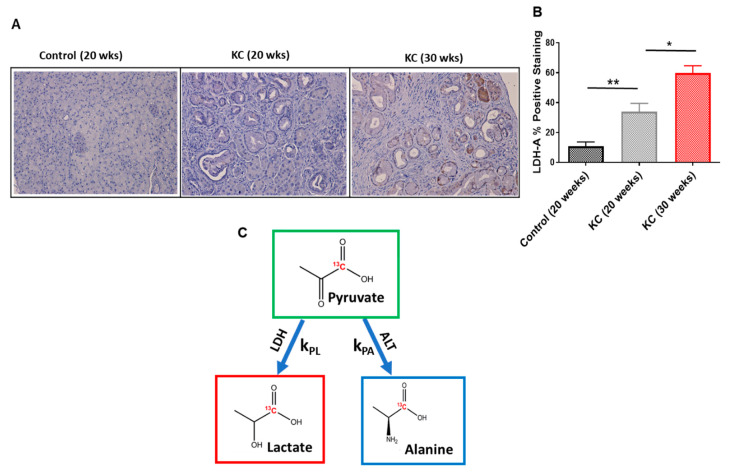
(**A**) Immunohistochemistry staining of LDH-A protein expression level for normal pancreas (control 20 wks) and early- (KC 20 wks) and advanced-stage (30 wks) PanINs. (**B**) Comparison of positive staining (%) scores among the tree different mice groups (*n* = 10). The percentage of LDH-A positive staining significantly increases as the PanINs progress to advanced stage. (**C**) A schematic of conversion kinetics of pyruvate to lactate and alanine and the corresponding conversion rate constants (k_PL_ and k_PA_). The PanIN progression significantly alters these enzymatic kinetics. * means *p* < 0.05; ** means *p* < 0.001.

We investigated whether there was a difference in the expression level of the LDH-A protein depending on the stages of PanINs. LDH-A was significantly overexpressed in advanced-stage PanINs (30 weeks) compared with early-stage PanINs (20 weeks; Figure 4A). We also quantified the score for positive staining of LDH-A, and it is statically significant between the two groups of mice (*p* = 0.035; Figure 4B).

## 3. Discussion

Molecular imaging techniques targeting changes in metabolism present exciting opportunities for detecting early stages of cancer. The successful application of HP-MRS to detect aggressiveness of the patient-derived pancreatic cancer xenografts has been reported recently by our group [28]. Grade dependent (low and high) prostate cancer demonstrates significant differences in lactate production, which is measurable in vivo via [1-^13^C] pyruvate-based HP-MRS [29]. It is worth noting that HP-MRS scans can be completed in 2–3 min, compared to 10–30 min of imaging times in other metabolic imaging modalities like FDG-PET.

The ability to accurately probe the metabolic phenotypes in vivo is essential to understand the nature of aggressive pancreatic cancer. The expression levels of LDH-A depend on the stages of PanINs. We also assessed the alteration of LDH and ALT enzymes activity with PanINs progression. Hyperpolarized pyruvate has been proven to be an important metabolic imaging probe, as the kinetic measurements of pyruvate-to-lactate and/or pyruvate-to-alanine conversion (Schematic Figure 4C) can predict the critical switching point from early-stage PanINs to advance PanINs that may correlate with transformation to PDAC. These alterations, known as metabolic reprogramming, provide a clear biochemical phenotype for detection and grading and may guide potential treatment and prevention options in the clinics. The metabolic switching as observed in this study, LDH actively dominating over ALT function, is a clear indication of PanINs progression to advanced stages. From a therapeutic intervention perspective, there will be a window of opportunity for inhibition of metabolic pathways to suppress the disease progression. At the same time, the clinical evaluation can be achieved by non-invasive HP-MRS.

HP-MRS is thus an efficient technique to interrogate reaction kinetics driven by metabolic enzymes in vivo. The metabolite ratios were used as indicators for biological processes in diseases and healthy status. In addition, it is desirable to quantify the reaction rate constants. The first-order rate constants, k_PL_ and k_PA_ of LDH and ALT mediated reactions, respectively, were extracted by modeling the signal intensity versus time data of hyperpolarized [1-^13^C] pyruvate, lactate, and alanine. The data suggest competitive enzyme activities between ALT and LDH in PanIN lesions, and LDH dominates over ALT when PanINs progress to advanced stages.

Our metabolic imaging study demonstrates the ability to assess altered metabolism applying HP-MRS and ^1^H-NMR spectroscopy to genetically engineered mouse models that develop different stages of PanINs with time. This offers us an opportunity to longitudinally monitor the progression of PanINs along with metabolic plasticity via real-time in vivo imaging. This result will drive the development of a novel imaging strategy for detection of preneoplastic lesions in high-risk individuals in a clinical setting. Imaging murine pancreas by MRI is challenging given its small size. Each lobe of pancreas consists of several smaller lobes called lobules. In humans, lobules measure 1–10 mm in diameter, whereas in mice, they are 0.5–1.5 mm in diameter [30]. We adopted a slab-selective approach to acquire HP-MRS data in this study. This approach has some limitations as described in the Results section. Our future study will employ more precise and localized ^13^C-HP-MRI methodology employing chemical shift imaging (CSI). Eventually, that approach may be translated in human subjects with high risk of developing pancreatic cancer. If successful, early detection could lead to earlier interventions at higher cure rates. Since a latency period of up to 15 years has been reported [31] for precursor lesions to malignant transformation in pancreatic cancer, screening of high-risk population may open a window of opportunity for significant outcome improvements.

## 4. Materials and Methods

### 4.1. Development of Genetically Engineered Mice

All animal experiments were conducted in compliance with the National Institutes of Health (NIH) guidelines for animal research and were approved by the Institutional Animal Care and Use Committee (IACUC) of the University of Texas MD Anderson Cancer Center. We used the conditional P48:Cre; LSL-KRAS^G12D^ (KC) mice, which were bred and maintained at our facility. We sacrificed mice (*n* = 10 for each group) at 10, 20, and 30 weeks of age, respectively, as described in the early work on PanINs [12]. As control, we used P48:Cre or WT C57BL/6 littermates sacrificed at the same age as experimental mice.

### 4.2. Histology and Quantification of PanINs Progression

Murine pancreatic tissue was fixed in 4% paraformaldehyde, embedded in paraffin, and cut into 5 μm sections. Tissue was then stained with H&E following standard protocols. We used ImageJ software for pancreatic lesions quantification as previously described [2,31]. Briefly, lesions were classified as either acinar-to-ductal metaplasia (ADM), early pancreatic intraepithelial neoplasia (PanINs), or advanced PanINs. The software calculated the percent of total tissue surface area occupied by each lesion for 8–10 random fields in each of the slides.

### 4.3. Immunohistochemistry

Paraformaldehyde-fixed, paraffin-embedded murine pancreatic tissue sections were deparaffinized and rehydrated. For antigen retrieval, tissue was boiled with 1X citrate buffer, pH 6.0 (Sigma), for 15 min. We then put slides in 3% H_2_O_2_ for 15 min to block endogenous peroxidases. Nonspecific epitopes were blocked with HyClone Bovine Serum Albumin (GE Healthcare Life Sciences) for 15 min. The sections were incubated overnight at 4 °C with LDH-A Antibody (Cell Signaling Technology). This was followed by an HRP-conjugated secondary antibody for 1 h. Then we applied Signal Stain DAB Substrate Kit (Cell signaling) following the manufacturer’s instructions. Finally, slides were counterstained with hematoxylin and mounted in Acrytol Mounting Medium (Electron Microscopy Sciences).

### 4.4. Lactate Dehydrogenase (LDH) and Alanine Aminotransferase (ALT) Enzymes Assay

Active LDH and ALT enzymes assays (colorimetric) (MAK066 and MAK052, Sigma) were performed using homogenized murine pancreatic tissue collected at 20 and 30 weeks of age. The assays were performed according to the manufacturer’s instructions. Murine tissue was homogenized with LDH or ALT buffer. Lysates were used to check for LDH or ALT activity, and samples were measured every 3-5 min for up to 60–90 min. The protein concentration of each homogenized tissue was measured by Quick Start Bradford Protein Assay (Bio-Rad) and normalized.

### 4.5. ^13^C Pyruvate Hyperpolarization

[1-^13^C] pyruvic acid was purchased from Isotec Sigma Aldrich (St. Louis, MO, USA). The OX063 trityl radicals were purchased from GE Healthcare, Amersham, Little Chalfont, UK. A solution of 25 μL of [1-^13^C] pyruvate, containing 15 mM of OX063 and 1.5 mM of chelated gadolinium (Magnevist, Bayer Healthcare, Wayne, NJ, USA), was polarized at 3.35T and 1.4 K in a HyperSense DNP polarizer (Oxford Instruments, Abingdon, UK). Each hyperpolarized sample was rapidly dissolved in 4.0 mL of a superheated alkaline dissolution buffer, containing 40 mM of 4-(2-hydroxyethyl)-1-piperazineethanesulfonic acid, 30 mM of NaCl, and 100 mg/L of ethylenediaminetetraacetic acid (EDTA). ^13^C- dynamic spectra were acquired right after the 80 mM hyperpolarized [1-^13^C] pyruvate solution was administrated via a tail vein catheter.

### 4.6. T_2_-weighted proton MRI

Conventional MRI and ^13^C MRS in vivo were performed on a 7T MRI scanner (Bruker Biospin MRI GmbH, Ettingen, Germany) with a dual tuned ^1^H/^13^C volume coil (ID: 35 mm, Doty Scientific Inc., Columbia, SC, USA). Proton anatomic images were taken using a multi-slice T_2_-weighted RARE (rapid acquisition with relaxation enhancement) sequence. Respiratory gating was adopted to minimize motional artifact during image acquisition. Images of different view/planes including axial, coronal, and sagittal were acquired to identify the best location of the PanINs or region of interest on the mouse pancreas. The imaging parameters of the T_2_-weighted scans were: echo time T_E_ = 15 ms, repetition time T_R_ = 2.0 s, field of view = 40 mm x 30 mm, 256 μm x 256 μm in-plane resolution, 10–12 1 mm slices, and four averages.

### 4.7. In Vivo ^13^C MRS

A series of slab-selective ^13^C spectra (thickness 4–5 mm on pancreatic lesions) were collected right after injection of hyperpolarized [1-^13^C] pyruvate using spFLASH sequence. Total of 90 transients were acquired with a time delay between each transient being 2 s (total time 3 min). Each transient used a 12 ° flip angle excitation pulse (gauss pulse) and 2048 data points. A small 8 M ^13^C-urea phantom doped with Gadolinium-DPTA was placed in each mouse experiment for chemical shift referencing. Data were processed in MATLAB (MathWorks Inc., Natick, MA, USA), TopSpin (Bruker BioSpin GmbH, Ettlingen, Germany), or MestReNova (Mestrelab Research, Santiago, Spain). The dynamic spectra were manually phased, and line-broadening was applied (10–12 Hz). The area under the spectral peaks for pyruvate and lactate were integrated over the whole array. The lactate-to-pyruvate metabolic flux ratios (Lac/Pyr) were calculated by taking the ratio over the sum of lactate and pyruvate signals [32]. Similarly, the Ala/Pyr ratio was assessed. The apparent rate constants k_PL_ (pyruvate to lactate) and k_PA_ (pyruvate to alanine) were determined using the unidirectional kinetic modeling [33,34,35] with further refinement as described in the Results section.

### 4.8. Ex Vivo ^1^H NMR Spectroscopy

PanINs nodules were excised and flash-frozen in liquid nitrogen. The frozen tissue samples were weighed, crushed, and immersed in 3 mL of methanol and water mixture (2:1) on top of 0.5 mL of polymer vortex beads inside a 15 mL test tube. A process of mechanical homogenization was performed by vortexing the tubes for 30 seconds, again flash-freezing in liquid nitrogen for 1 min, and allowing the mixture to thaw. This process was repeated three times. The samples were then subjected to centrifugation for 10 min to separate the water-soluble metabolites from the proteins and other cellular constituents. The supernatant was extracted and subjected to rotary evaporation to remove the methanol. The samples were further desiccated by placing them on a lyophilizer overnight, leaving just the collection of metabolites. The metabolites were then immersed in a solution of 600 μL of D_2_O, 36 μL of K_2_HPO_4_ buffer, and 4 μL of 80 mM TMSP [3-trimethylsilyl)-1-propanesulfonic acid-d_6_ sodium salt]. The phosphate buffer was added to stabilize any potential pH variations, and the TMSP served as the reference standard to which we normalized the spectral signal from each metabolite. All supplies (D_2_O, TMSP, phosphate buffer) were purchased from Sigma-Aldrich and used without further purification.

NMR spectra were obtained using a Bruker AVANCE III HD^®^ NMR scanner (Bruker BioSpin MRI GmbH) at a temperature of 298 K. The spectrometer operates at a ^1^H resonance frequency of 500 MHz and is endowed with a triple resonance (^1^H, ^13^C, ^15^N) Prodigy BBO cryogenic temperature probe with a Z-axis shielded gradient. A pre-saturation technique was implemented for water suppression. The spectra were obtained with a 90 ° pulse width, a scan delay t_rel_ of 6.0 s, a 10,240 Hz spectral width, and an acquisition time t_max_ of 1.09 s (16,000 complex points). A total of 256 scans were collected and averaged for each spectrum, which resulted in a total scan time of 32 min and 49 seconds. Here, t_rel_ + t_max_ was nearly 8 s, so that it was greater than 3 × T_1_ of the metabolites observed. The time domain signal was apodized using an exponential function.

After the spectra were acquired, metabolic profiling was performed in Chenomx NMR Suite 8.1 software (Chenomx Inc., Edmonton, Canada). Quantification of the metabolites was then performed using MestReNova software (Mestrelab Research, A Coruña, Spain) by integrating some nonzero region centered about the chemical shift at which the metabolite is known to resonate. This integral value is then normalized by the value of the integral of the TMSP reference peak [28].

### 4.9. Statistical Method

Experimental values reported are means ± SD (standard deviation). Statistical significance among the animal groups was assessed by using a two-sample *t*-test assuming unequal variances and plotted using Graph-Pad Prism software (GraphPad Software, La Jolla, CA, USA). Statistical significance was considered at the *p* value < 0.05 level where *p* value is probability value.

## 5. Conclusions

We demonstrated that hyperpolarized metabolic imaging is capable of separating the metabolic signatures of benign and aggressive pancreatic cancer precursor lesions. The ability to monitor the progression non-invasively and the management of high-risk patients can be achieved by HP-MRS. This technique may have the potential to identify a window of therapeutic opportunity in which emerging therapeutic interventions might be applied to the high-risk pancreatic cancer subjects. The research described here has the potential for leading to practice-changing recommendations for non-invasively detecting and monitoring advanced PanIN lesions and incipient pancreatic cancer.

## Figures and Tables

**Table 1:** Real-time in vivo metabolic flux data obtained by HP-MRS.

Mice Group (*n* = 10)	Ala/Pyr	Lac/Pyr	Kinetic Constant k_PA_, (s^−1^)	Kinetic Constant k_PL_, (s^−1^)
Control (20 weeks)	0.195 ± 0.004	0.261 ± 0.003	0.0098 ± 0.0005	0.0125 ± 0.0005
KC (20 weeks)	0.132 ± 0.005	0.452 ± 0.002	0.0076 ± 0.0006	0.0224 ± 0.0004
KC (30 weeks)	0.096 ± 0.006	0.643 ± 0.003	0.0055 ± 0.0005	0.0296 ± 0.0004

Alanine-to-pyruvate ratio (Ala/Pyr) and the kinetic constant k_PA_ values decrease as the PanINs progress to advanced stages. In contrast, lactate-to-pyruvate ratio (Lac/Pyr) and the kinetic constant k_PL_ values increase in advanced stages of PanINs.

## Supplementary Materials

The following are available online at https://www.mdpi.com/1422-0067/21/10/3722/s1, Kinetic Modelling.
