# Supplementary material for: Early Detection of Pancreatic Intraepithelial Neoplasias (PanINs) in Transgenic Mouse Model by Hyperpolarized 13C Metabolic Magnetic Resonance Spectroscopy
Source: Int J Mol Sci. 2020 May 25;21(10):3722. doi: 10.3390/ijms21103722 (PMC7279395; doi:10.3390/ijms21103722)
Supplement: Supplementary file 1 [file ijms-21-03722-s001.pdf]

International Journal of *Molecular Sciences*

# Kinetic Modeling

A precise function was used to estimate the level of pyruvate over time. The function is of form:

$$f(t) = \begin{cases} 0 & t < t_s \\ \frac{r_p}{k_p}\left(1 - e^{-k_p(t-t_s)}\right) & t_s \le t < t_e \\ f(t_e)e^{-k_p(t-t_e)} & t \ge t_e \end{cases}$$

where $r_p$, $k_p$, $t_s$, and $t_e$ are estimated by minimizing the error sum of squares.

After estimation of these parameters, we estimate the alanine-to-pyruvate and lactate-to-pyruvate ratios over time. The fitted functions are of the form:

$$g(t) = \begin{cases} 0 & t < t_s \\ \frac{k_{px} r_p}{k_p - k_x}\left(\frac{1 - e^{-k_x(t-t_s)}}{k_x} - \frac{1 - e^{-k_p(t-t_s)}}{k_p}\right) & t_s \le t < t_e \\ \frac{f(t_e)k_{px}}{k_p - k_x}\left(e^{-k_x(t-t_e)} - e^{-k_p(t-t_e)}\right) + g(t_e)e^{-k_x(t-t_e)} & t \ge t_e \end{cases}$$

where $x$ represent alanine and lactate, and $k_{px}$, $k_x$ are estimated by minimizing the error sum of squares. The fitted plots are shown in the article Figure 3 C and the parameters are listed in Table 1

© 2020 by the authors. Submitted for possible open access publication under the terms and conditions of the Creative Commons Attribution (CC BY) license (http://creativecommons.org/licenses/by/4.0/).
